# Human Saliva-Mediated Hydrolysis of Eugenyl-β-D-Glucoside and Fluorescein-di-β-D-Glucoside in In Vivo and In Vitro Models

**DOI:** 10.3390/biom11020172

**Published:** 2021-01-27

**Authors:** Mariusz Dziadas, Adam Junka, Henryk Jeleń

**Affiliations:** 1 Faculty of Chemistry, University of Wrocław, 50-383 Wrocław, Poland; 2 Department of Pharmaceutical Microbiology and Parasitology, Medical University of Wrocław, 50-556 Wrocław, Poland; adam.junka@umed.wroc.pl; 3 Faculty of Food Science and Nutrition, Poznan University of Life Science, 60-624 Poznań, Poland; henrykj@up.poznan.pl

**Keywords:** β-glucosides, hydrolysis, human saliva

## Abstract

Eugenyl-β-D-glucopyranoside, also referred to as Citrusin C, is a natural glucoside found among others in cloves, basil and cinnamon plants. Eugenol in a form of free aglycone is used in perfumeries, flavourings, essential oils and in medicinal products. Synthetic Citrusin C was incubated with human saliva in several in vitro models together with substrate-specific enzyme and antibiotics (clindamycin, ciprofloxacin, amoxicillin trihydrate and potassium clavulanate). Citrusin C was detected using liquid chromatography with tandem mass spectrometry (LC-MS/MS). Citrusin C was completely degraded only when incubated with substrate-specific *A. niger* glucosidase E.C 3.2.1.21 (control sample) and when incubated with human saliva (tested sample). The addition of antibiotics to the above-described experimental setting, stopped Citrusin C degradation, indicating microbiologic origin of hydrolysis observed. Our results demonstrate that Citrusin C is subjected to complete degradation by salivary/oral cavity microorganisms. Extrapolation of our results allows to state that in the human oral cavity, virtually all β-D-glucosides would follow this type of hydrolysis. Additionally, a new method was developed for an in vivo rapid test of glucosidase activity in the human mouth on the tongue using fluorescein-di-β-D-glucoside as substrate. The results presented in this study serve as a proof of concept for the hypothesis that microbial hydrolysis path of β-D-glucosides begins immediately in the human mouth and releases the aglycone directly into the gastrointestinal tract.

## 1. Introduction

Knowledge concerning potential mechanism of natural release of biologically active aglycone by microbial enzyme hydrolysis of β-D-glucosidic bond from the paternal glucoside molecule on human tongue is extremely scanty, as well as about rapid methods for determining β-glucosidase activity in the oral cavity. Aglycones are non-sugar part of glucosides with positive health properties like quercetin however, can also be harmful as in case of deoxynivalenol. It has been thought that hydrolysis of dietary β-glucosides (flavonoids and isoflavones) takes place in the large intestine due to the colon microflora [1]. However, metabolites of some flavonols and isoflavones appear in plasma within 30 min of ingestion, indicating rapid absorption in the small intestine [2,3]. In one study [4], it was shown that the cell-free extracts form human small intestine and liver had β-glucosidase activity towards flavonoid and isoflaflavone glycosides. Data presented by other authors included the hydrolysis of rutin in cell-free extracts collected from human fecal flora and saliva [5]. Hydrolysis of several other flavonoid glycosides by microbiota was also reported [6]. In work [7], it was shown that deoxynivalenol-3-glucoside (D3G) was hydrolyzed to deoxynivalenol during digestion, indicating the microbial origin of the hydrolysis process, as the D3G was known to be resistant to acids and enzymes expressed by humans. Likewise, in work [8], atmospheric pressure ionization–mass spectrometry (API-MS) was successfully applied to detect hexanol released in mouth from hexyl β-D-glucoside consumed. Also in work [9], it was shown that naturally odorless glucosides of fruits, when incubated with microorganisms, release active odorant molecules. The rapid confirmation of a microorganism’s ability to hydrolyze β-D-glycosidic bond in the human oral cavity may be of paramount meaning with regard to its potential applications in early medical diagnostics, pharmacy, biotechnology and food processing due to aglycons liberated from glucosides, which are molecules with several biological activities. The microbe-induced hydrolysis of β-D-glycosidic bond may find a broad spectrum of application wherever active substance release or targeted aglycone delivery is needed. Therefore, the aim of this research was to confirm oral microorganisms’ ability to hydrolyze β-D-glycosidic bond directly on the tongue.

## 2. Materials and Methods

The following materials were used in this work: eugenol 99%, β-D-glucose pentaacetate 98%, acetic anhydride 99%, hydrogen bromide (32% solution in acetic acid), anhydrous chloroform, anhydrous dichloromethane, anhydrous toluene, anhydrous ethyl acetate, anhydrous diethyl ether, anhydrous methanol, anhydrous isopropanol, anhydrous sodium sulfate, β-D-glucosidase from *A. niger* (~750 U/g), potassium hydroxide, sucrose 99.5%, sodium methoxide solution (MeONa) 25 wt.% in methanol, Dowex resin 50WX8H, clindamycin hydrochloride, ciprofloxacin 98%, amoxicillin trihydrate: potassium clavulanate (4:1), fluorescein di-β-D-glucopyranoside (FbD), HMDS 99%, and anhydrous Na_2_SO_4_. In addition, trifluoroacetic acid anhydrous (spectroscopic grade) was purchased from Sigma Aldrich, Poland. SiO_2_ 60 TLC plates were purchased from Merck-Millipore, Poland. Silver oxide (Ag_2_O) of analytical grade was purchased from Chempur, Poland.

### 2.1. A New In Vivo Rapid Method for the Detection of β-D-Glucosidase Activity in the Oral Cavity

The authors chose FbD as a reaction indicator because the aglycon fluorescein is a non-toxic substance also used in medicine. Analysis of the fluorescein under UV light gave representative results in the form of a yellow color, which contrasts with the blue background of a UV light (254 nm). Other glycosides in the determination of enzymatic activities are known, such as 4MU-β-D-Glucoside used in Gaucher disease as an indicator, or the more toxic PNP-β-D-glucoside. 

The release of free aglycone in the oral cavity was recorded as the proof of principle. 50 µL of commercially available FbD solution (1 mg/mL, deionized water) was used to soak a 1-cm square sterile paper filter. Then, the paper squares were placed on the center of tongue base of healthy volunteer. The series of pictures under UV light was taken every 15 s for 2 min. The aim of this proof of principle was to record shining of released fluorescein which confirms hydrolysis of β-D-glycosidic bond and this same β-D-glucosidase activity.

### 2.2. Two Kinetics of In Vitro Hydrolysis of FbD with A. niger β-D-Glucosidase and Human Saliva

The 1 mL solution of glucosidase from *A. niger* (14 mg/10 mL sterile deionized water) and 1 mL of solution of FbD in sterile deionized water (1 mg/mL) was placed into plastic cuvette and very gently mixed and placed into UV-VIS spectrophotometer for kinetic measurement (Thermo Evolution 300, Thermo Sci., USA) at 37 °C with the wavelength set to 480 nm.

Solution of FbD in sterile deionized water (5 mg/mL) was mixed with a solution of fresh saliva diluted with sterile deionized water 1:10, *v*/*v* (saliva was taken from two healthy volunteer males before lunch) at a proportion of 1 mL solution FbD and 1 mL solution of diluted saliva. Two mL of these test solution was pipetted into a plastic cuvette and placed into a UV-VIS spectrophotometer for kinetic measurement (Thermo Evolution 300, Thermo Sci., USA) at 37 °C where the wavelength was set 480 nm.

### 2.3. Incubation of Selected Pathogens Strains with Fluorescein-Di-β-D-Glucoside (FbD)

All strains: SAW-*Staphylococcus aureus* ATCC 6538, CAW-*Candida albicans* ATCC 10231, CEW-*Escherichia coli* ATCC 25922, FEW-*Enterococcus faecalis* ATCC 29212, PAW-*Pseudomonas aeruginosa* ATCC 15442, STRES-*Streptococcus sanguinis* ATCC 10556, STREM-*Streptococcus mutans* ATCC 25,175 were cultured in Tryptone Soya Broth (Argenta, Poland) in an incubator (Advantage-Lab 6, VWR, Poland) at 37 °C/5CO_2_. Next, the strains’ density (CFU) was established using a densitometer (Densilameter^®^ II densimeter; Erba Lachema, Brno, Czech Republic) to 0.5 McFarland and then, using a serial dilution method, to a density of 10^5^ colony-forming units/mL. Subsequently, 100 µL of such microbial suspension was transferred to the wells of a 96-well plate with 50 µL FbD solution 1 mg/mL. All plates was shaken in a Vortex 96-well plate at 37 °C (Biosan, Józefów, Poland). Plates was analyzed on a Multiskan UV-VIS spectrophotometer (Thermo Sci., Waltham, USA) at a 480 nm wavelength.

#### Microbial Species Isolation from Kefir Product and Preparation of Microbial Protein Extract for MALDI-TOF

A loopful of kefir (KR1) product was cultured into appropriate media-Saboroud, MRS Lab Agar, BHI agar (Biocorp, Poland) and incubated for 24–72 h at 28 °C or 37 °C in microbiological incubator (Binder, VWR, Poland). After incubation, the reductive culturing was performed in order to obtain distinct microbial colonies. Incubation was repeated analogically. Next, each distinct microbial colony was introduced to Eppendorf tube containing 300 µL of deionized water and subjected to thorough vortex-mixing. Afterwards, 900 µL of pure ethanol was added to the tube. The vortex-mixing was repeated. Subsequently, tube was centrifuged under 14,000 rpm/min for 2 min at 4 °C. Supernatant was removed and then 20 µL of 70% formicide was added to the pellet and vortex-mixed again. Next, 20 µL of pure acetonitrile was added and centrifugation stage was repeated in analogical manner to this described-above. Obtained supernatant was collected and subjected to the MALDI-TOF analysis.

The identification of microorganisms using MALDI-TOF technique was performed on UltrafleXtreme (Bruker Daltonics, Bremen, Germany) mass spectrometer. Ca. 1 uL of each prepared protein extract was spotted on MALDI target (MTP 384 Polished Steel, Bruker), left to dry on air and covered with 1 uL of matrix solution (HCCA, 20 mg/mL solution in water:acetonitrile: TFA 50:47.5:2.5 (*v*/*v*/*v*)). Bacterial Test Standard (Bruker Daltonics, Bremen, Germany), prepared according to producer’s guidelines, was used as a calibrant. The measurements were performed in positive ion mode with linear detection. Method crucial setups were as follows: mass range 2000–20,000 *m*/*z*, laser power 30%, collected laser shots 1000–2000 (depending of the aquired spectra intensity). Mass spectra were processed and identified with MALDI Biotyper 3.0 software (Bruker Daltonics, Bremen, Germany) with built-in spectral microorganism database consisting of ca. 4600 records. Protein extract of previously identified E.coli strain served as positive control of experiment.

### 2.4. Organic Synthesis of Eugenyl β-D-Glucoside

Pentaacetate-β-D-glucose (2 g) was mixed with 20 mL of HBr and 5 mL of acetic anhydride in ice cooled round bottom flask for 3 h. After reaction (TLC SiO_2_, toluene: ethyl acetate 2:1, *v*/*v*) products were dissolved in 50 mL of chloroform and vigorously washed with 4 portions of deionized water (4 × 100 mL each). Organic layer was separated and dried over anhydrous sodium sulfate (all night at 8 degrees Celsius) and afterward, the filtration solvent was evaporated using a rotary evaporator. To crude syrup 5 mL anhydrous diethyl ether was added and after 1 min, gentle mixing white crystals of acetylated glucose bromide was observed. If a crystallization problem occurs, then 1 mL of petroleum ether can be added if necessary. The product was dried for 1 h on glass funnel in vacuum flow and stored under argon in dark vial at −18 °C.

**Scheme 1:**
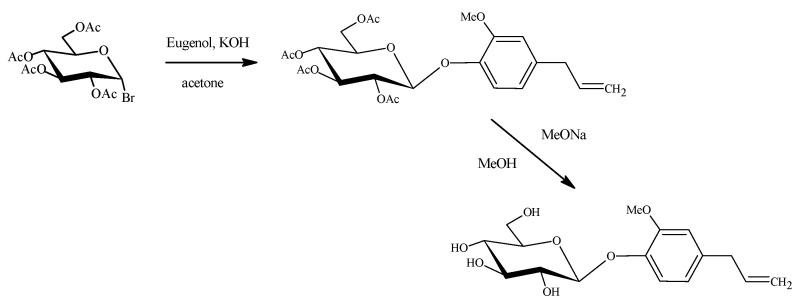
Scheme of organic synthesis eugenyl β-D-glucoside (Citrusin D). Molecular mass Citrusin D after synthesis using HR-MS (Figure S1).

A solution of glucosyl bromide (5 mmol) in anhydrous acetone (20 mL) was slowly added through glass syringe to thoroughly mixed (1000 rpm) solution of eugenol (15 mmol) in 1.0 mol/L potassium hydroxide (10 mL) and the solution was stirred for 3 h at room temperature. The completion of reaction was monitored by TLC (SiO_2_, toluene: ethyl acetate 3:1, *v*/*v*). After reaction the was complete, acetone was removed by evaporation under vacuum using rotary evaporation. The resulting suspension was extracted with anhydrous dichloromethane (4 × 50 mL). The crude product in dichloromethane was washed with 11% potassium hydroxide (6 × 30 mL), water (6 × 40 mL), and dried by anhydrous sodium sulphate (all night in 8 Celsius degree). After filtration and removal of the solvent using rotary evaporation, the crude product (eugenyl pentaacetate-β-D-glucopyranoside) was recrystallized from anhydrous isopropyl alcohol as light brown crystals (Scheme 1 Table 1). The final product was dried using funnel with a glass filter under vacuum.

The peracetylated glucosides (0.6 mmol) were solubilized in a solution of MeONa in anhydrous MeOH (20 mL, 1.0 mol/L) and stirred at room temperature for 30 min. After the completion of the reaction, noticed by TLC (SiO_2_, chloroform:methanol 4:1, *v*/*v*), the mixture was neutralized with resin Dowex 50WX8H. The resin was filtered off and washed with methanol (2 × 5 mL). The collected filtrate was concentrated in vacuum to afford pure eugenyl β-D-glucoside. (Scheme 1).

### 2.5. Experiment of Hydrolysis Eugenyl β-D-Glucoside In Vitro with Antibiotics

#### 2.5.1. Testing of Biological Activity of Substrate 

10 mg artificial glycoside was placed into a 2 mL vial and 1 mL solution β-D-glucosidase from *A. niger* (2 mg/mL) was added. After 5 min of reaction, 50 µL of this solution was pipetted to a 2 mL vial and 800 µL dry piride was added with an internal standard (D-sorbitol, 2 mg/mL) and 100 mg anhydrius sodium sulfate. All solution was mixed on vortex and 200 µL was taken to empty 2 mL vial and 200 µL HMDS (hexamethyldisilazane) and 50 µL anhydrous TFA acid. The solution was mixed on a vortex and after 5 min, the reaction of the upper phase was taken to clear 300 µL insert and analyzed on a GC/MS system. A QP2010-Ultra GCMS was used for analysis (Shimadzu, Japan) using capillary column ZB-5 ms (30 m × 0.25 × 0.25 ID) Phenomenex, USA. Injector temp. was 300 °C, split 1:30 and oven program: 100 °C-hold 1 min, then rate 10 °C/min to 290 °C and hold during 10 min. Analysis was carried out in scan mode at 33–700 amu. The spectra was analyzed using GCMSolution ver.4.2 (Shimadzu, Japan) with NIST 2017 spectra library.

#### 2.5.2. Preparation of Reactions

For this experiment, saliva was collected from two of the authors (25 min before lunch, did not eat or drink anything before collection of saliva, so enzymes of only microbiota, not of transient microorganisms, were present in a material taken), spit once directly into sterile glass beaker (250 mL) and redissolve with sterile water (Milli-Q) 1: 10 *v*/*v*. All solutions were prepared in water, Citrusin D (2.5 mg/mL), β-D-glucosidase from *A. niger* (2 mg/mL), solution of all antibiotic (2 mg/mL each), 2% sucrose. As a reactor for each experiment, 20 mL of headspace sterile vials was used with a Teflon membrane screw cap. Each vial was been capped immediately after dosage (*n* = 3). Dosage of individual solution is shown in Table 1. 

#### 2.5.3. Sample Preparation and LC-MS/MS Analysis of Citrusin D

0.5 mL of each sample was taken through a septa using a 1 mL syringe and slowly filtered through 0.2-µm syringe filter (Whatman) directly to a 2 mL autosampler vial with a 300 µL conical insert (Alwsci Technologies). All samples were prepared and collected in an autosampler, with a temperature 4 °C of an HPLC system in 24 and 48 h after start of experiment. 

For the detection of Citrusin D, a Shimadzu Prominence UFLC coupled with Shimadzu 8030 triple quadrupole mass detector was used. Chromatographic separation was performed on a Phenomenex Kinetex Synergi Fusion, reversed phase column C-18 (100 × 2 mm, 2.5 µm) at flow rate 1.0 mL/min with a mobile phase A, which consisted of 0.1% formic acid in water and mobile phase B consisted of 0.1% formic acid in acetonitrile. The following gradient elution was used: 0 min-10% B, 7 min-100% B. The column thermostat and autosampler temperatures were maintained at 30 °C and 5 °C, respectively. Sample volume injected into the system was 10µL. MRM-MS/MS parameters: precursor ion (*m*/*z*) 371.0, fragment ions (*m*/*z*) 148.1, dwell time (msec.) 20, collision energy (V) 38, collision gas pressure (kPa) 230, desolvation line temp. (°C) 250, heat block temp. (°C) 400, nebulizing gas flow (mL) 2, drying gas flow (2 mL) 10. Instrument control and data analysis were carried out by Shimadzu Lab Solution software ver. 5.65.

## 3. Results

### 3.1. A New In Vivo Rapid Method for the Detection of β-D-Glucosidase Activity in the Oral Cavity on the Tounge

**Figure 1:**
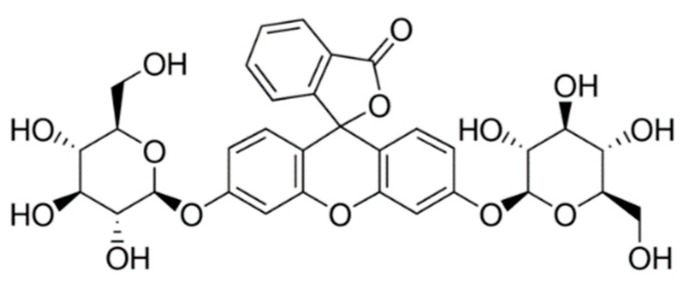
Chemical structure of fluorescein di-(β-D-glucopyranoside)-FbD.

**Figure 2:**
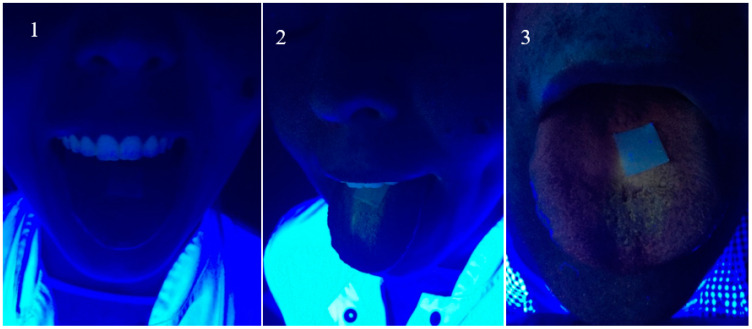
Proof of the principle showing microbial-induced hydrolysis of glucosidic β-D-bond. Picture 1: fluorescein glycoside-soaked paper is placed on the tongue surface. Picture 2: liberation of fluorescein resulting in shining of a UV light after 15 s of incubation on the tongue surface. Picture 3: Fluorescein detection (in 20 s.) liberated from glucoside after being washed in mouth three times with a mixture of water with Octenisept 1:1, *v*/*v*.

The ability of human saliva to hydrolyze glucoside bonds was proven by applying fluoresceine glycoside (Figure 1) soaked paper on a tongue surface. A commercial, non-shining DfB was placed on the tongue and left for 2 min. Figure 2 shows the results of in-mouth hydrolysis fluorescein di-β-D-glucoside with released fluorescein monitored under a UV light of 254 nm.

### 3.2. Kinetics In Vitro Hydrolysis of FbD with Enzyme and Saliva

**Figure 3:**
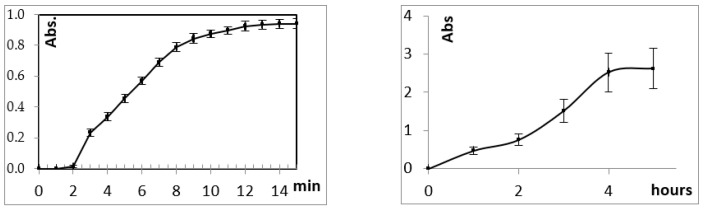
Kinetics of FbD degradation by the β-D-glucosidase enzyme from *A. niger* (480 nm, 37 °C)-left curve. Kinetics of FbD decomposition in diluted human saliva-right curve (480 nm, 37 °C).

The assessment of the FdB glycoside hydrolysis capacity and the hydrolysis kinetics are shown in Figure 3. The eft graph is the kinetics with the *A. niger* β-D-glucosidase—a typical course of an enzymatic reaction. Conversion to 8 min., which occurs quickly, is observed and is then followed by a flattening of the kinetic curve due to the inhibition of the reaction by glucose. The right graph shows the kinetics of FbD hydrolysis in diluted human saliva. A picture of cuvette from the experiment (Figure S2).

### 3.3. Incubation of Selected Pathogens Strains with FbD

**Figure 4:**
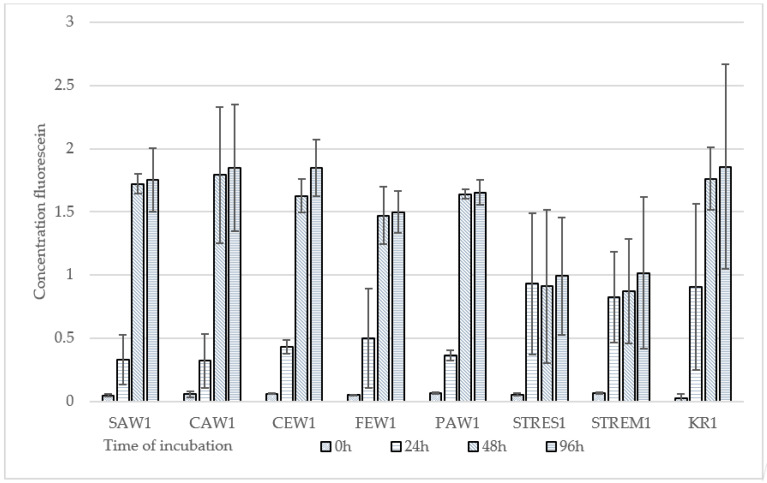
Changes in the concentration of dehydrolyzed fluorescein aglycone during the incubation of selected microorganisms with FdB. SAW- *Staphylococcus aureus*, CAW-*Candida albicans*, CEW-*Escherichia coli*, FEW-*Enterococcus faecali*, PAW-*Pseudomonas aeruginosa*, STRES-*Streptococcus sanguinis*, STREM-*Streptococcus mutans*. KR1-sample of kefir commercially avaible in local store. Typical looks from experiment 96-well plate (Figure S2).

In order to confirm the ability of selected bacteria to hydrolyze the glucosidic bond, the standard strains were incubated with the substrate (FbD) using 96-well plates in the experiment. Changes in FbD concentration were monitored as an increase in dehydrolysed fluorescein at 480 nm. Figure 4 shows the change in FdB content over the 96 h of observation.

During the observation, the high activity of FbD hydrolyzing by the sample containing k.efir was noted. The microbiological analysis of the applied kefir product revealed presence of two dominating microorganisms within this product, namely: *Lactobacillus plantarum* and *Sacharomyces cerevisiae*. Matrix-Assisted Laser Desorption/Ionization-Time Of Flight (MALDI-TOF) analysis confirmed the results of the microbiological analysis, where the best match was *Saccharomyces cerevisiae* (score value 2.204) and *Lactobacillus brevis* (score value 2.365). The full report from Biotyper (Figure S3).

### 3.4. Organic Synthesis of Eugenyl β-D-Glucoside

The synthesis of eugenyl-β-D-glucopyranoside was performed as presented in Materials and Methods, Section 2.4. 4-allyl-2-methoxyphenyl b-D-glucopyranoside as a light brown crystals were obtained in a yield of 47%. The identity of synthesized compound was confirmed using NMR; Bruker Avance III 500 MHz, number of scan 32, relaxation delay 1 s., aquistion time 2.62 s., 30 degree flip angle, size of FID 65536; and the following signals were obtained for it: ^1^H NMR (MeOD, 500 MHz, 298 K) δ ppm: 7.02 (d, 1H, 3j = 8.2 Hz, Ar-H), 6.76 (d, 1H, 4j = 2.0 Hz, Ar-H), 6.66 (dd, 1H, 3j = 8.2 Hz, 4j = 2.0 Hz, Ar-H), 5.83–5.84 (m, 1H), 5.05–4.92 (m, 3H, OH), 4.79–4.77 (m, 1H, OH), 3.82–3.75 (m, 5H, CH), 3.65–3.60 (m, 1H, CH), 3.44–3.20 (m, 8H, CH), ^13^C NMR (MeOD, 125 MHz) d ppm: 149.3(1C, ar.), 144.9 (1C, ar.), 137.6 (1C, allylic), 135.1 (1C, ar.), 120.6 (1C, ar.), 116.8 (1C, allylic), 114.4 (1C, ar.), 112.8 (1C, ar.), 101.7 (1C, sugar), 76.8 (1C, sugar), 76.5 (1C, sugar), 73.6 (1C, sugar), 69.9 (1C, sugar), 61.1 (1C, sugar), 55.3 (1C, sp3), 39.4 (1C, sp3); Mol. formula: C16H22O7; Mol. Weight: 326.24. The synthesized glycoside was used subsequently in experiments. NMR spectra ^1^H NMR and ^13^C NMR (Figures S4 and S5).

#### Synthetic Glycoside Biological Activity Test

**Figure 5:**
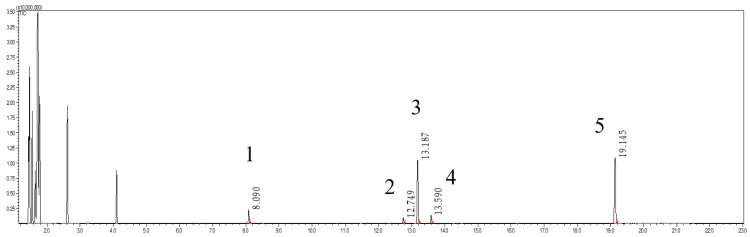
Chromatogram obtained from analysis of derivatized sample from hydrolytic suspension with enzyme *A. niger* after 4 min of starting the reaction. Peaks 1-Eugenol TMS deriv., 2-α-D-glucose-5-TMS deriv., 3-D-sorbitol-6-TMS deriv., 4-β-D-glucose-5-TMS deriv., 5-eugenyl β-D-glucoside-4-TMS deriv.

Before using the glycoside as a substrate, a test for biological activity and beta configuration was performed. Analysis performed used method described in 2.5.1. Chromatogram from analysis was shown on Figure 5. Characteristic mass spectra for peaks (Figure S6).

### 3.5. Experiment of Hydrolysis Eugenyl β-D-Glucoside In Vitro with Human Saliva and Antibiotics

**Figure 6:**
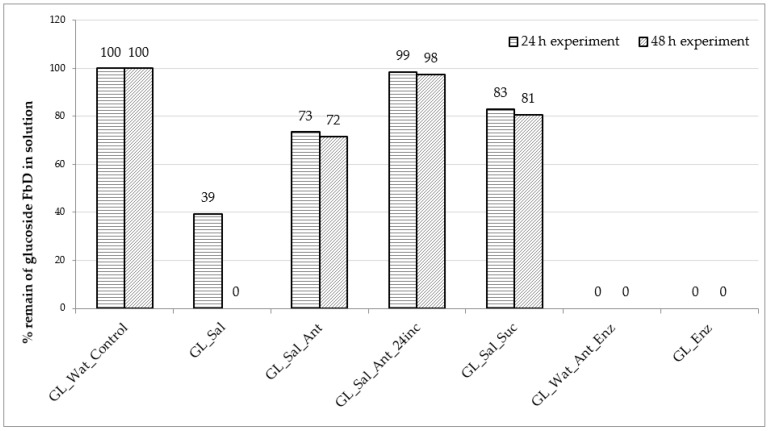
Changes [%] in the concentration of FbD during incubation in test solution. GL_Wat_Control-Citrusin D + water, GL_Sal-Citerusin D + saliva solution, GL_Sal_Ant-Citrusin D + saliva solution + mix of antibiotics, GL_Sal_Ant_24inc.-Citrusin D + saliva solution + mix of antiobiotic solution after incubation for 24 h Citrusin D was added, GL_Sal_Suc-Citrusin D + saliva solution + sucrose solution, GL_Wat_Ant_Enz-Citrusin D + water + mix of antibiotiocs + enzyme *A. niger*, GL_Enz-Citrusin D + water + enzyme *A. niger*.

The changes in glycoside concentration during incubation with human saliva in different sequences were measured by LC-MS/MS. Below (Figure 6) are the results of the observation of the entire experiment.

## 4. Discussion

The aim of the work was to explain the phenomenon of the hydrolysis of the β-D-glucosidic bond in the human oral cavity on the tongue. Previous reports indicated the activity of peroxidase enzymes from saliva [10] where the used substrate was onion soup and remnants of quercetin and its glycoside were detected, which hydrolyze in saliva during 4 h. This 4 h period of time is the same as the time on Figure 3, when FbD with saliva was monitored and after 4 h termination of aglycone concentration increase was noted. Berthiller’s work clearly indicated the possible involvement of microorganisms in the hydrolysis of the β-D-glycosidic bond when cellobiase from *Aspergillus* (70% yield in 18 h) and *Lactobacillus plantarum* (62% yield in 8 h) was used as the hydrolytic source. 

The fact that hydrolysis is taking place on the tongue and not in the saliva directly when the substrate appears on the tongue is the basis of the proposed FbD rapid test. FbD contain two beta-D-glucose moieties, and almost instantaneous dehydrolysed fluorescein was observed after 15 s of substrate exposure to the tongue. Similar results obtained in [8] when APCI–MS was used for measure hexanol release from hexyl b-d-glucoside in the mouth. The sample was introduced at 15 s and the first signal from MS was observed around 30 s of exhaling air into the spectrometer. The porosity of the tongue is so great that it is an excellent habitat for billions of microorganisms that can degrade the glucosidic bond very quickly. Even rinsing the mouth with a disinfectant did not affect the enzymatic activity on the tongue. This indicates a very large accumulation of the active ingredient on the tongue with glucosidase activity and kinetic reactions (Figure 3-left) with the enzyme *A. niger* compared to diluted saliva indicate the microbial origin of the glucosidase activity. The main feature is a much slower rate of hydrolysis when in enzyme reaction after 8 min was observed to finish degradation when 4 h was needed for stopping the degradation with the solution of saliva. 

In fact, the residence time of food in the oral cavity is shorter than in the intestines and it therefore seems that the pre-digestion processes is likely proportionally faster to the intestinal processes. The experiment where FbD was incubated with selected pathogens in an optimal environment shows that the beginning of the hydrolysis process occurred in the first 24 h (Figure 4). The hydrolytic transformations on the tongue take place dynamically within 15–30 s (Figure 2). This may be due to a different qualitative composition of the oral microflora from intestine. Incubation with selected microorganisms shows that they have the ability to break down the β-D-glucosidic bond. This is shown in Figure 5 where fluoresceine as a reaction product was monitored. The strongest FdB was hydrolyzed by *S. aureus*, *C. albicans* and by a mixed culture with *S. cerevisiae* and *L. brevis* kefir and the slowest degradations of FbD was observe by *S. mutans* and *S. sanguinis*. All of the tested strains showed the ability to hydrolyze FbD, which is associated with the enzymatic apparatus for obtaining the carbon source in the first day of the logarithmic growth phase. 

The second proof of hydrolysis by microorganisms shows the experiment with eugenol glycoside. Especially vial 4 *, which contained previously incubated saliva with antibiotics where in the same sample, a glycoside loss of 30% was observed, but without prior incubation with the antibiotic. Antibiotics did not affect the activity of the enzyme itself, which indicates that they affect the source from which the enzyme is produced, i.e., the bacterial cell. Sample with sucrose solution was 20% glucoside loss caused probably sucrose retained the enzymatic activity of microorganisms by being a better carbon source.

Eugenol is a natural substance which found a variety of industrial, gastronomical and medicinal applications especially in dental medicine when is used as a cement component for tooth canals (cloves oil). However, when bound to glycoside, it is devoid of its biological activity. 

In this paper, for the first time, it was demonstrated that eugenyl β-D-glucoside may be hydrolyzed by oral cavity microorganisms resulting in release of free eugenol aglycone form. Extrapolation of our results may suggest that not only eugenyl β-D-glucoside, but virtually all glycosides possessing a β-D-bond, may undergo a similar hydrolysis process when incubated with oral cavity microorganisms. Such a discovery may be broadly applied wherever active substance release or targeted flavor delivery is needed especially when recurrent pulpitis is observe caused by *S. mutans*, which has the possibility to liberated aglycone from glucosidic bond (Figure 5).

The line of investigation presented in this research paper has no analogies in previously published articles, therefore a comparison of our findings to the existing literature is problematic and cannot be performed in the scope wider than this performed by us in the Introduction [1,2,3,4,5]. The generalization of the phenomena would require a higher number of investigated glycosides. However one should bear in mind that majority of these compounds are commercially unavailable and need to be synthesized by researchers themselves. This research was focused on the development of a simple and rapid method for the detection glucosidase activity on the tongue and on analysis of eugenyl-β-D-glucoside, when eugenol is in the form of a free aglycone, can be used in a vast variety of applications. 

Therefore, hydrolysis of eugenyl β-D-glycoside and the detection of free eugenol and a new rapid test FbD under a 254 nm light source could be treated as a strong proof of the principle confirming oral microorganisms ability to break the above-mentioned bond. 

## 5. Conclusions

We conformed the hypothesis that the glucosidic bond may be hydrolyzed by microorganisms on the tongue with test results. The rapid FbD hydrolysis test on the tongue also clearly confirms this hypothesis. The phenomenon of hydrolysis of beta glucoside bonds on the tongue can be widely used in medical diagnostics, pharmacy, and food technology as a mechanism for releasing active aglycone from the glycoside molecule.

## Figures and Tables

**Table 1:** Dosage (mL) of individual solution to sterile vials (V1–V8). V5 *-VIAL number 5 contains: saliva + antibiotics and after 24 of incubation 37 °C and then glucoside addition.

#	Water Solutions	V1	V2	V3	V4 *	V5	V6	V7
1	Saliva with water (1:10, *v*/*v*)		8	8	8	8		
2	Citrusin D (2.5 mg/mL)	1	1	1	1	1	1	1
3	β-D-glucosidase (2 mg/mL)						1	0.1
4	all antibiotics (2 mg/mL)			1	1		1	
5	sucrose (2%)					1		
6	sterile deionized water	9	1				7	9

## Data Availability

Not applicable.

## Supplementary Materials

The following are available online at https://www.mdpi.com/2218-273X/11/2/172/s1, Figure S1: Molecular mass Citrusin D after synthesis using HR-MS, Figure S2: A picture of cuvette from the experiment, Figure S3: Full report form Biotyper, Figure S4: ^1^H NMR spectrum, Figure S5: ^13^C NMR spectrum. Figure S6: GC/MS spectrum of investigated compounds.
