# Supplementary material for: Human Saliva-Mediated Hydrolysis of Eugenyl-β-D-Glucoside and Fluorescein-di-β-D-Glucoside in In Vivo and In Vitro Models
Source: Biomolecules. 2021 Jan 27;11(2):172. doi: 10.3390/biom11020172 (PMC7911702; doi:10.3390/biom11020172)
Supplement: Supplementary file 1 [file biomolecules-11-00172-s001.zip › Supplementary Fig.3 biotyper.pdf]

Bruker Daltonik MALDI Biotyper Classification Results

Project Info:

Project Name:	**Bruker Project**
Project Description:	Bruker Project Description
Project Owner:	Admin
Project Creation Date/Time:	6/18/2018 2:53:40 PM
Project Analyte Count:	3
Project Type:	Development
Validation:	not present
Validation Position:	

Result Overview

Analyte Name	Organism (best match)	Score Value	Organism (second best match)	Score Value
SAB (++) (A)	Candida_robusta[ana]# (Saccharomyces_cerevisiae[teleo])	2.204	Candida_robusta[ana]# (Saccharomyces_cerevisiae[teleo])	2.177
20180618 std BTS (++) (A)	Escherichia coli	2.1	Escherichia coli	2.076
Beetl (+++) (A)	Lactobacillus brevis	2.365	Lactobacillus brevis	2.335

Meaning of Score Values

Range	Description	Symbols	Color
**2.300 ... 3.000**	**highly probable species identification**	**(+++)**	green
**2.000 ... 2.299**	**secure genus identification, probable species identification**	**(++)**	green
**1.700 ... 1.999**	**probable genus identification**	**(+)**	yellow
**0.000 ... 1.699**	**not reliable identification**	**(-)**	red

Meaning of Consistency Categories (A - C)

Category	Description
**A**	**Species Consistency:** The best match was classified as 'green' (see above). Further 'green' matches are of the same species as the first one. Further 'yellow' matches are at least of the same genus as the first one.
**B**	**Genus Consistency:** The best match was classified as 'green' or 'yellow' (see above). Further 'green' or 'yellow' matches have at least the same genus as the first one. The conditions of species consistency are not fulfilled.
**C**	**No Consistency:** Neither species nor genus consistency (Please check for synonyms of names or microbial mixture).

Analyte1

BRUKER

Analyte Name:	SAB
Analyte Description:	D:\Data\Biotyper\20180618 KK dla AC\SAB\0_D9\1\1SLin
Analyte ID:	b2403596-e3f7-4e10-bfb1-bfe568f5a999
Analyte Creation Date/Time:	6/18/2018 12:52:25 PM
Applied MSP Library(ies):	
Applied Taxonomy Tree:	Bruker Taxonomy

Rank (Quality)	Matched Pattern	Score Value	NCBI Identifier
1 (++)	Candida_robusta[ana]# (Saccharomyces_cerevisiae[teleo]) INVSc1 BRL	2.204	4932
2 (++)	Candida_robusta[ana]# (Saccharomyces_cerevisiae[teleo]) WS LLH	2.177	4932

3<br>(++)	Candida_robusta[ana]# (Saccharomyces_cerevisiae[teleo]) BJ2168 BRL	2.13	4932
4<br>(++)	Candida_robusta[ana]# (Saccharomyces_cerevisiae[teleo]) DTY3 BRL	2.077	4932
5<br>(+)	Candida_robusta[ana]# (Saccharomyces_cerevisiae[teleo]) Isolat LGL Muenchen	1.991	4932
6<br>(-)	Candida_robusta[ana]# (Saccharomyces_cerevisiae[teleo]) Kontrollstamm Humanmedizin VML	1.648	4932
7<br>(-)	Candida_robusta[ana]# (Saccharomyces_cerevisiae[teleo]) 991400574 LBK	1.608	4932
8<br>(-)	Lodderomyces elongisporus CBS 2605T CBS	1.277	36914
9<br>(-)	Rhizobium radiobacter B165 UFL	1.221	359
10<br>(-)	Photobacterium iliopiscarium DSM 9896T HAM	1.217	56192

Analyte2

Analyte Name:	20180618 std BTS
Analyte Description:	D:\Data\Biotyper\20180612 KK dla BD\20180618 std BTS\0_D7\1\1SLin
Analyte ID:	436695e7-b5b5-4585-9aa1-236ed88d915a
Analyte Creation Date/Time:	6/18/2018 12:46:10 PM
Applied MSP Library(ies):	
Applied Taxonomy Tree:	Bruker Taxonomy

Rank (Quality)	Matched Pattern	Score Value	NCBI Identifier
1 (++)	Escherichia coli ATCC 25922 THL	2.1	562
2 (++)	Escherichia coli ATCC 35218 CHB	2.076	562
3 (++)	Escherichia coli ATCC 25922 CHB	2.052	562
4 (++)	Escherichia coli MB11464_1 CHB	2.045	562
5 (++)	Escherichia coli DH5alpha BRL	2.026	562
6 (+)	Escherichia fergusonii DSM 13698T HAM	1.906	564
7 (+)	Escherichia albertii DSM 17582T HAM	1.902	208962
8 (+)	Escherichia coli ESBL_EA_RSS_1528T CHB	1.897	562
9 (+)	Escherichia coli DSM 30083T HAM	1.845	562
10 (+)	Escherichia coli RV412_A1_2010_06a LBK	1.81	562

RUO

Analyte3

Analyte Name:	Beetl
Analyte Description:	D:\Data\Biotyper\20180618 KK dla AC\Beetl\0_D8\1\1SLin
Analyte ID:	b97abd95-0612-4748-ace5-63a78d8790ee
Analyte Creation Date/Time:	6/18/2018 12:51:10 PM
Applied MSP Library(ies):	
Applied Taxonomy Tree:	Bruker Taxonomy

Rank (Quality)	Matched Pattern	Score Value	NCBI Identifier
1 (+++)	Lactobacillus brevis DSM 20054T DSM	2.365	1580
2 (+++)	Lactobacillus brevis DSM 2647 DSM	2.335	1580
3 (+++)	Lactobacillus brevis DSM 1267 DSM	2.324	1580
4 (++)	Lactobacillus brevis DSM 20556 DSM	2.272	1580
5 (++)	Lactobacillus brevis DSM 1268 DSM	2.136	1580
6 (-)	Morganella morganii (PX) 22086121 MLD	1.26	582
7 (-)	Morganella morganii ssp sibonii Mb19277_2 CHB	1.232	180435
8 (-)	Lactobacillus acidifarinae 108702 CIP	1.222	267364
9 (-)	Clostridium beijerinckii 1072_ATCC 25752T BOG	1.203	1520
10 (-)	Proteus vulgaris (PX) 22086129 MLD	1.142	585

RUO
