# Supplementary material for: Human Saliva-Mediated Hydrolysis of Eugenyl-β-D-Glucoside and Fluorescein-di-β-D-Glucoside in In Vivo and In Vitro Models
Source: Biomolecules. 2021 Jan 27;11(2):172. doi: 10.3390/biom11020172 (PMC7911702; doi:10.3390/biom11020172)
Supplement: Supplementary file 1 [file biomolecules-11-00172-s001.zip › Supplementary Fig.5 13CMD1.pdf]

SpinWorks 4: user MOB

C13CPD1h MeOD {C:\-\mob} {NMRLAB\auto} 1

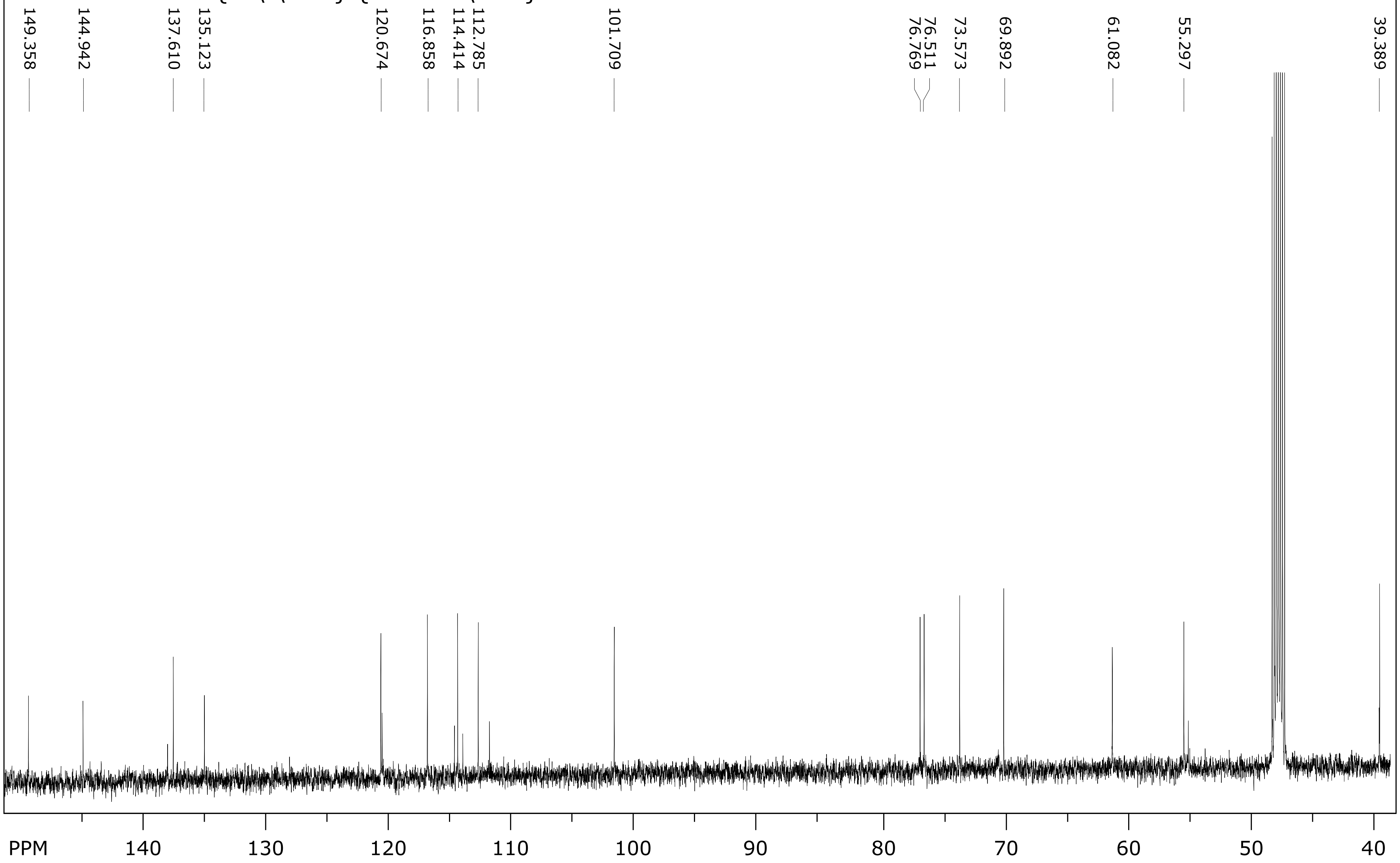

file: C:\nmr\MD1\2\fid expt: <zgpg30>
transmitter freq.: 125.770364 MHz
time domain size: 65536 points
width: 29761.90 Hz = 236.6369 ppm = 0.454131 Hz/pt
number of scans: 1024

freq. of 0 ppm: 125.757788 MHz
processed size: 32768 complex points
LB: 1.000 GF: 0.0000
Hz/cm: 568.122 ppm/cm: 4.51713
