# Supplementary material for: Human Saliva-Mediated Hydrolysis of Eugenyl-β-D-Glucoside and Fluorescein-di-β-D-Glucoside in In Vivo and In Vitro Models
Source: Biomolecules. 2021 Jan 27;11(2):172. doi: 10.3390/biom11020172 (PMC7911702; doi:10.3390/biom11020172)

## Supplemental GC/MS spectra

Eugenol – TMS deriv.

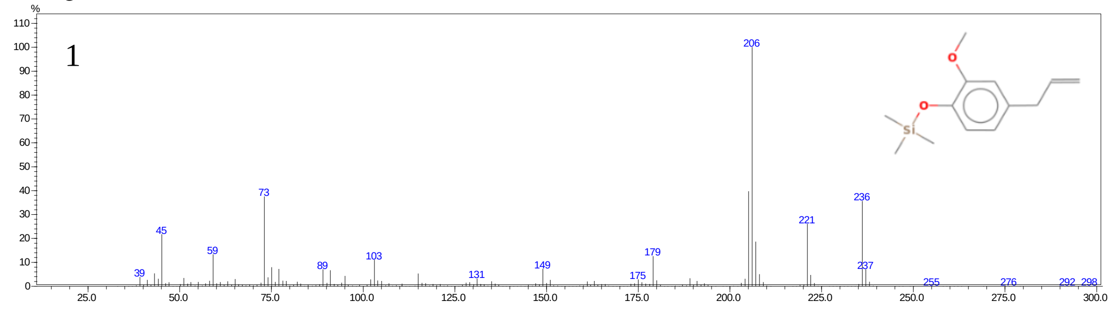


*α*-D-glucose – 5 TMS deriv.

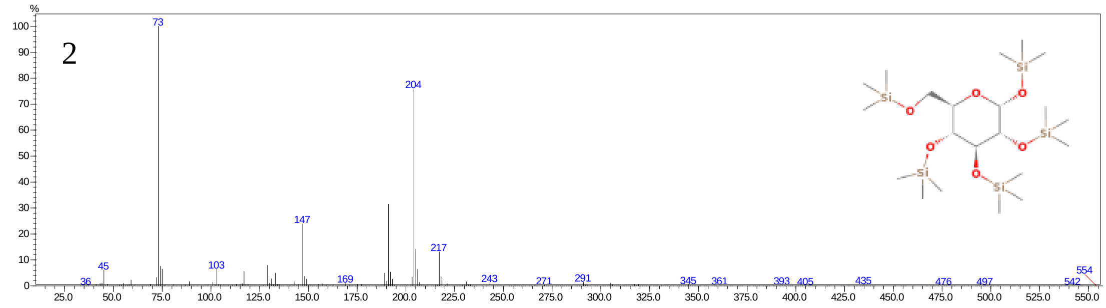


*D-sorbitol – 6 TMS deriv.*

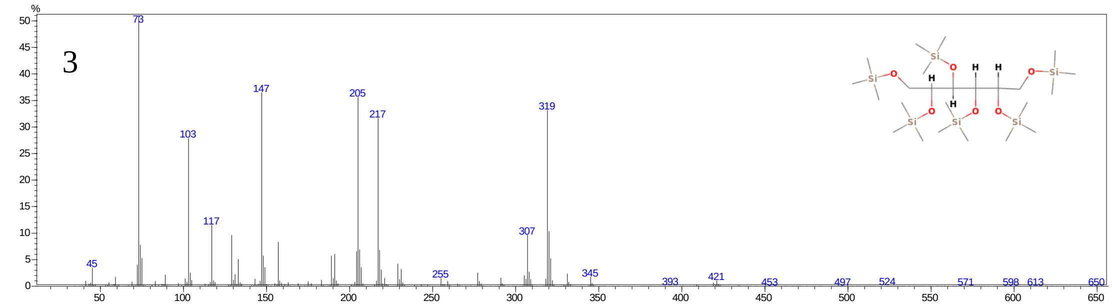


*β-D-glucose 5 TMS deriv.*

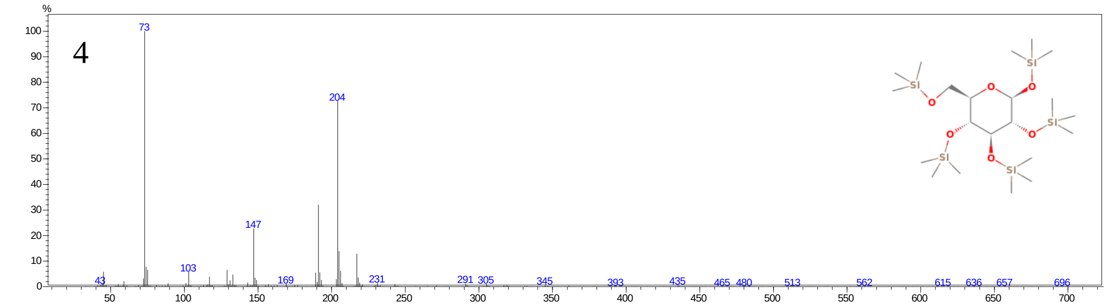


*Cytrusin D – 4 TMS deriv.*

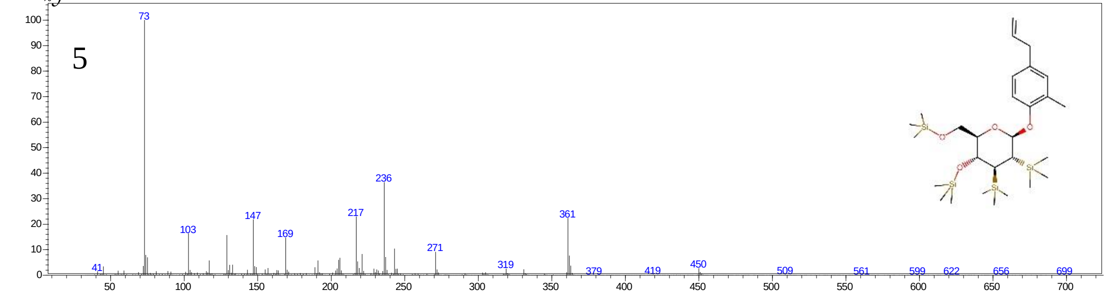

Supplement: Supplementary file 1 [file biomolecules-11-00172-s001.zip › Supplementary Fig.6.pdf]
