# Supplementary material for: Human Saliva-Mediated Hydrolysis of Eugenyl-β-D-Glucoside and Fluorescein-di-β-D-Glucoside in In Vivo and In Vitro Models
Source: Biomolecules. 2021 Jan 27;11(2):172. doi: 10.3390/biom11020172 (PMC7911702; doi:10.3390/biom11020172)
Supplement: Supplementary file 1 [file biomolecules-11-00172-s001.zip › Supplementary Fig.2.pdf]

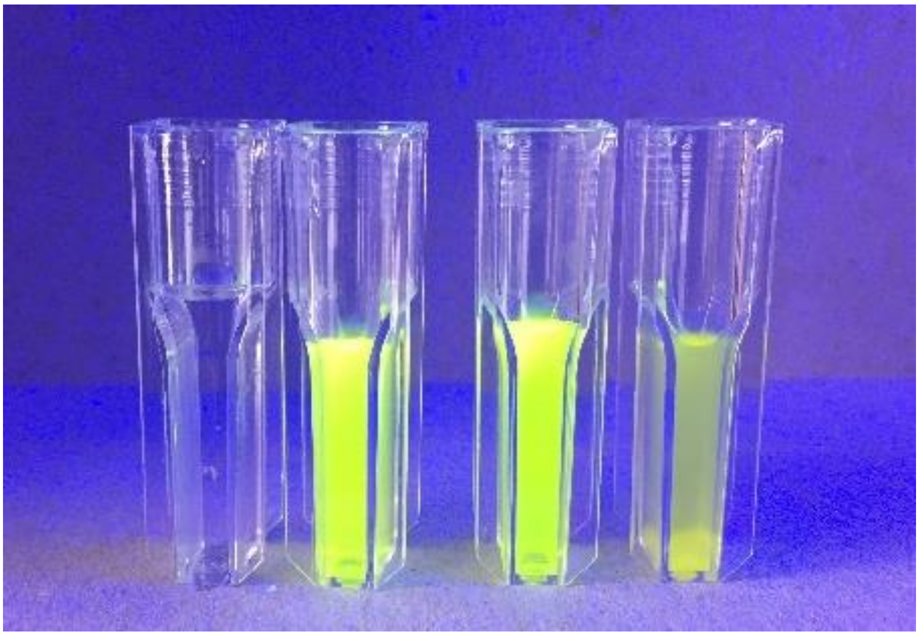

Picture of cuvette used in kinetric reaction after finish – from left : zero sample water, enzyme from A. niger, enzyme from sweet almonds, sample with saliva solution

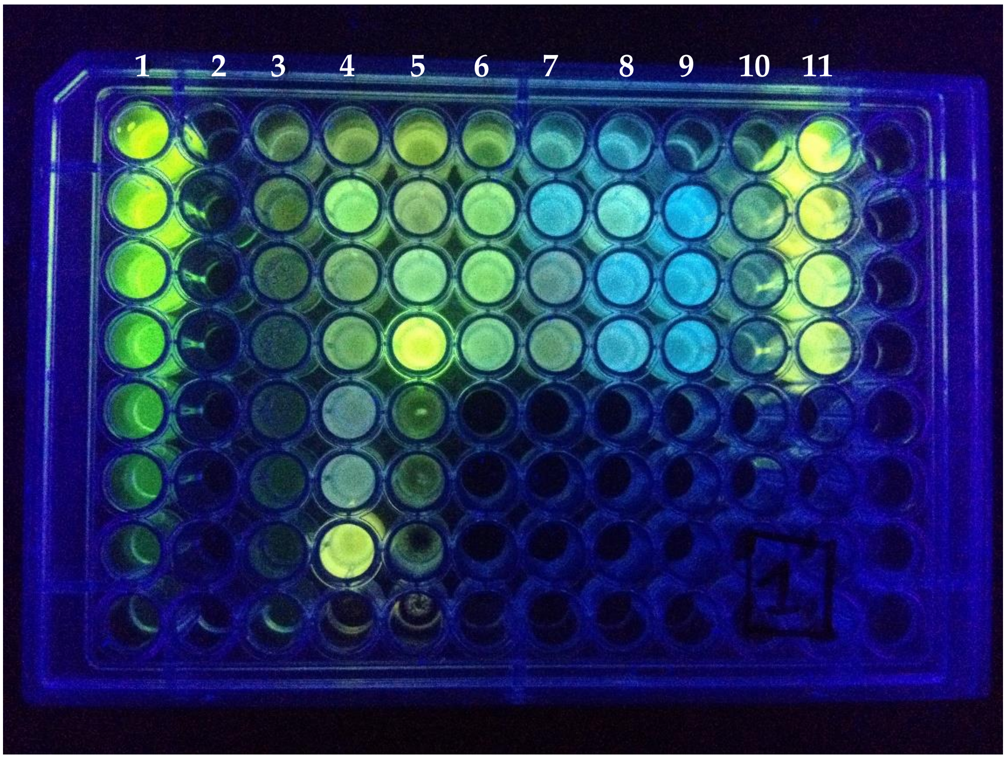

One 96-well plate from plates during experiment with bacterias
