# Supplementary figures and images for: Human Saliva-Mediated Hydrolysis of Eugenyl-β-D-Glucoside and Fluorescein-di-β-D-Glucoside in In Vivo and In Vitro Models
Source: Biomolecules. 2021 Jan 27;11(2):172. doi: 10.3390/biom11020172 (PMC7911702; doi:10.3390/biom11020172)

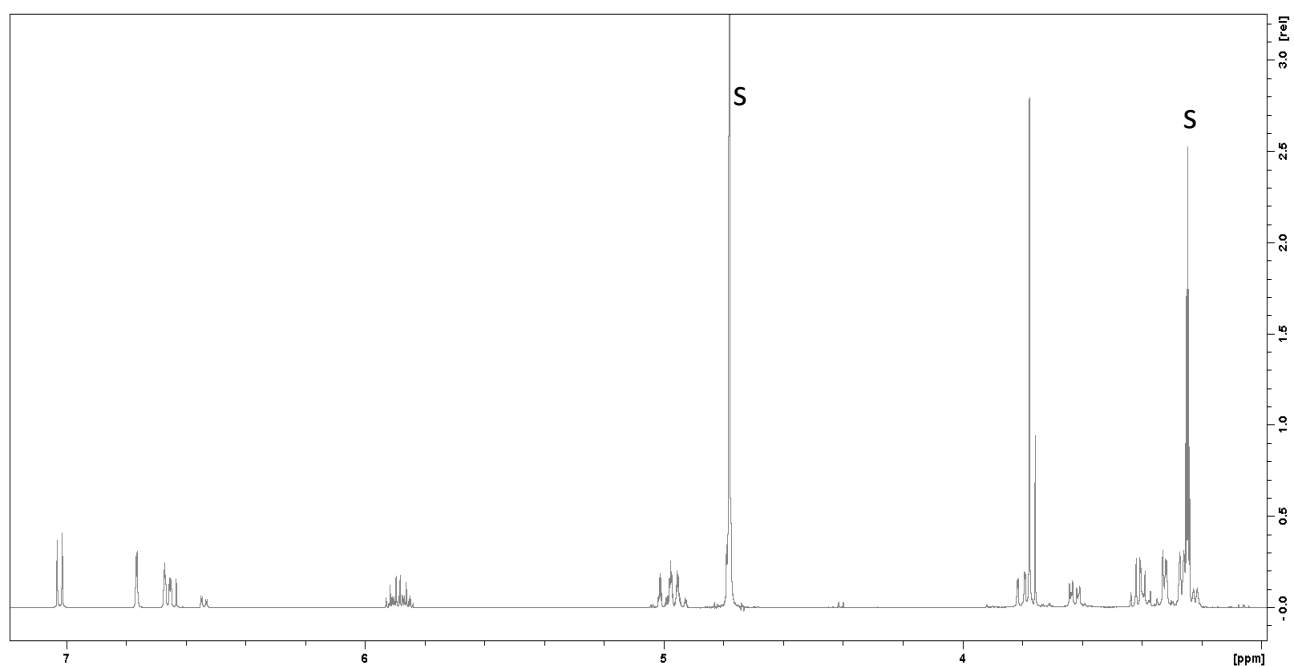

$^1\text{H}$  NMR spectrum of eugenyl- $\beta$ -D-glucopyranoside (500 MHz, methanol- $d_4$ , 298 K); s – solvent

Supplement: Supplementary file 1 [file biomolecules-11-00172-s001.zip › Supplementary Fig.4 1HNMR.pdf]

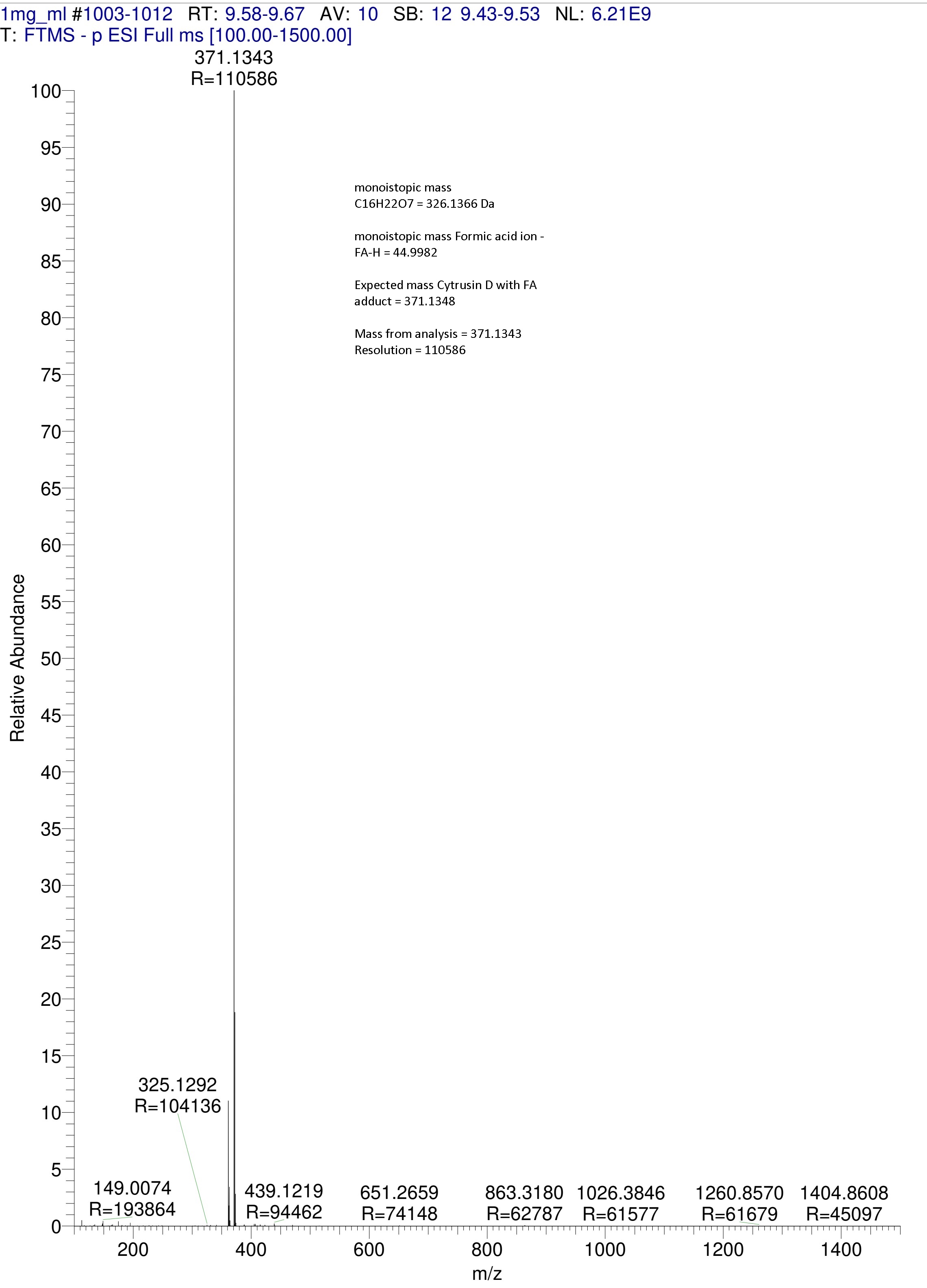

Supplement: Supplementary file 1 [file biomolecules-11-00172-s001.zip › Supplementary Fig.1.jpg]
